# Associations between neonatal brain structure and neurodevelopmental outcomes following very preterm birth

**DOI:** 10.1038/s41372-026-02672-3

**Published:** 2026-04-15

**Authors:** Zeyuan Sun, Yan Ge, Marguerite Leoni, Andrew T. M. Chew, Andrew J. Lawrence, Serena J. Counsell, Joseph V. Hajnal, A. David Edwards, Paola Dazzan, Dafnis Batalle, Chiara Nosarti

**Affiliations:** 1 Department of Child and Adolescent Psychiatry, Institute of Psychiatry, Psychology and Neuroscience, King’s College London, London, United Kingdom; 2 Research Department of Early Life Imaging, School of Biomedical Engineering & Imaging Science, King’s College London, London, United Kingdom; 3 Department of Forensic and Neurodevelopmental Science, Institute of Psychiatry, Psychology & Neuroscience, King’s College London, London, United Kingdom; 4 Department of Psychological Medicine, Institute of Psychiatry, Psychology, and Neuroscience, King’s College London, London, United Kingdom; 5 National Institute for Health Research (NIHR) Mental Health Biomedical Research Centre at South London and Maudsley NHS Foundation Trust and King’s College London, London, United Kingdom

**Keywords:** Predictive markers, Risk factors, Medical imaging, Paediatrics

## Abstract

**Background:**

Very preterm (VPT) infants face an elevated risk of neurodevelopmental delays, yet early predictors of specific impairments are poorly understood. This study investigated how neonatal brain structure relates to neurodevelopmental delays in VPT toddlers.

**Methods:**

We analysed term-equivalent T2-weighted MRI scans from 352 VPT infants. Neurodevelopmental outcomes were assessed at 18–24 months using the Bayley Scales of Infant and Toddler Development-III. We used tensor-based morphometry to compare voxel-wise whole-brain volumes between infants with and without developmental delay.

**Results:**

Toddlers with motor delays showed significantly reduced volume in the left posterior cerebellum at term compared to those without motor delays, even after adjusting for other domains. No significant volumetric differences were observed for cognitive or language delays.

**Conclusion:**

Reduced cerebellar volume at term is associated with motor delay in VPT toddlers. These findings highlight the cerebellum’s key role in early motor development and the value of structural MRI for early risk stratification.

## Introduction

Infants born very preterm (≤32 weeks’ gestation) are at greater risk of neurodevelopmental delays, including cognitive, language, and motor impairments, as well as behavioural problems [1, 2]. These delays, which may persist into adolescence and even adulthood, could limit individuals’ ability to engage in both structured and informal learning and social opportunities, thereby affecting their overall life outcomes [3, 4]. Although various screening tools have been developed to detect neurodevelopmental delays following very preterm birth [5, 6], reliable early predictors of non-optimal outcomes remain poorly understood, representing a significant ongoing clinical challenge.

Cognitive, language, and motor delays associated with very preterm birth may be partly explained by underlying structural and functional brain alterations. Since very preterm birth occurs during the third trimester of gestation, which represents a critical stage of brain growth, it may disrupt key neurodevelopmental processes, thereby increasing the risk of later developmental delays. Numerous studies have explored the associations between neonatal brain features and subsequent neurodevelopmental outcomes in very preterm infants [7]. For example, multimodal neuroimaging markers, including larger cortical volume, higher fractional anisotropy, and lower subcortical mean diffusivity, of preterm infants were associated with enhanced cognitive outcomes at 18–24 months [8]. Conversely, reduced volumes in subcortical regions, such as the basal ganglia and thalamus, have been associated with poorer motor development [8]. Recent studies also revealed an association between stronger structural cortico-striatal and thalamo-striatal connectivity at term and improved cognitive and language developmental outcomes at 18 months [9], whereas we previously showed that larger neonatal insular and orbitofrontal volumes characterised very preterm children with more favourable neurocognitive outcomes at 4 [7]. Furthermore, prior studies have tended to investigate how brain structure and function relate to individual developmental outcomes (e.g., cognition, language, or motor skills) [10], or to study older children [11]. Consequently, the potential associations between neonatal brain alterations and the co-occurrence of multiple developmental delays (e.g., cognitive, language, and motor) in the very first years of life following very preterm birth remain poorly understood.

Both prospective and retrospective analytic approaches have been widely employed in neuroimaging research to examine brain–behaviour relationships. Some studies have classified infants based on neuroimaging features to explore associations with later outcomes [12, 13], whereas others have grouped participants based on developmental outcomes and retrospectively examined earlier brain structures [14, 15]. The latter approach is particularly valuable for identifying early neurobiological markers associated with clinically meaningful developmental outcomes. Exploring early structural brain alterations linked to distinct developmental profiles could directly inform clinical practice through early identification of at-risk infants. Moreover, this method may better account for sample heterogeneity, enabling the detection of neonatal brain alterations that may underlie atypical developmental paths.

Using tensor-based morphometry (TBM), this study aimed to explore the shared and unique regional neonatal brain alterations at term associated with cognitive, language, and motor developmental delays in very preterm toddlers. We hypothesised that very preterm toddlers with developmental delays would display structural brain alterations at term compared to those without developmental delays. We further hypothesised that multiple domains of neurodevelopmental delays would share structural brain alterations and explored whether there were specific brain regions which were uniquely associated with delay in one developmental domain.

## Methods

### Study design and population

Participants were 511 very preterm infants enrolled in the Evaluation of Preterm Imaging study (ePrime, EudraCT: 2009-011602-42) [16]. Infants were recruited at birth between April 2010 and July 2013 from 14 hospitals within the North and South-West London Perinatal Network. Infants recruited into ePrime had the following inclusion criteria: birth before 33 weeks of gestation, maternal age above 16 years, and mothers not being hospital inpatients. Exclusion criteria were major congenital malformations, contraindications to magnetic resonance imaging (MRI), parents not being able to speak English, or being subject to child protection proceedings. Parents of 485 infants consented their offspring to undertake MRI at term-equivalent age (38–44 weeks’ postmenstrual age; PMA) and follow-up neurodevelopment assessments at 18–24 months of age. Our analyses included a subsample of 352 participants with quality neonatal MRI who completed follow-up neurodevelopmental assessments (detailed exclusion criteria in Supplementary Appendix 1). The ePrime study was conducted under the ethical standards of the 1964 Helsinki Declaration and was approved by the Hammersmith and Queen Charlotte’s Research Ethics Committee (09/H0707/98).

### MRI acquisition

Infants underwent MRI at term-equivalent age using a 3-Tesla system (Philips Medical Systems, Best, The Netherlands) with an 8-channel phased array head coil. During MRI, infant care was supervised by a paediatrician and included continuous monitoring of pulse oximetry, temperature, and electrocardiography data. Ear protection was provided with silicone-based putty (President Putty, Coltene Whaledent, Mahwah, NJ, USA) and neonatal earmuffs (MiniMuffs, Natus Medical Inc., San Carlos, CA, USA). 87% of infants were scanned under sedation (25–50 mg kg⁻¹ oral chloral hydrate) in accordance with institutional protocol, when they were unable to maintain natural sleep or exhibited agitation. High-resolution anatomical images were obtained by T2-weighted fast spin echo sequences (repetition time = 8670 ms; echo time = 160 ms; flip angle = 90°, slice thickness = 1 mm, field of view = 220*220 mm^2^, voxel size = 0.86*0.86*1 mm^3^, total scan time = 58 min).

### Structural data processing

Following the methods described in ref. [14] and ref. [15], T2-weighted images were registered to a study-specific template using Advanced Normalization Tools (ANTs) software Symmetric Normalisation algorithms [17]. Following rigid and affine image transformations, nonlinear transformations were used to generate deformation tensor fields within the template space and eliminate global volume differences. The resulting tensor fields describing the voxel-wise shape and volume changes from the template to each subject image were used to calculate log-transformed scalar Jacobian determinants. Smoothing with 4 mm full-width half-maximum Gaussian filter was applied.

### Socio-demographic and clinical measures

The following socio-demographic and clinical measures were collected as part of ePRIME: assigned sex at birth, maternal education (categorised as “low” if she left full-time education before the age of 19, otherwise as “high”) [18], birth weight, gestational age at birth (in weeks), infant’s PMA at scan, and age at follow-up assessment. We also included relative social deprivation, indexed by the 2010 Index of Multiple Deprivation (IMD), which was calculated based on maternal postcode at recruitment [19].

### Neurodevelopmental measures

Psychomotor development in toddlers was assessed using the Bayley Scales of Infant and Toddler Development–Third Edition (BSID-III) [20] at 18–24 months of age. Scores from its three subscales (cognitive, language, and motor development) were used for analysis.

### Statistical analysis

Statistical analysis was performed using R-4.3.1 (R Project for Statistical Computing). Descriptive results for categorical variables were presented as *n* (%) and continuous variables as median [interquartile range]. 40 participants with major brain lesions (defined as cystic periventricular leukomalacia, more than ten punctate white matter lesions, and/or grade 3 or 4 germinal matrix hemorrhage) evaluated by a senior radiographer [21] were also excluded. Participants included for imaging analysis (*n* = 352) were compared to those with major brain lesions to ensure the representativeness of the sample. The remaining participants had longer gestational age than those with major brain lesions (W = 8155.5, *p* = 0.01). However, there were no statistically significant differences between lesion groups in terms of sex distribution (χ = 0.33, *p* = 0.56), PMA at scan (W = 6316, *p* = 0.76), IMD (W = 6414, *p* = 0.72), and age at assessment (W = 6345, *p* = 0.80). According to the BSID-III scoring criteria, scores below 85 indicate developmental delay in the corresponding domain [22]. Therefore, participants were categorised into three pairs of groups: cognitive delay/non-delay, language delay/non-delay, and motor delay/non-delay. Clinical, demographic, and neurodevelopmental measures were compared between each pair of delayed and non-delayed groups using the Mann–Whitney U and Chi-Square tests (Table 1). We also described the correlation among the three BSID-III subscales and the comparisons of the proportion of delayed cases among BSID-III subscales in Supplementary Appendix 3 (Supplementary Tables 1, 2).

**Table 1:** Participants' characteristics (*N* = 352).

Variable	Summary measures
Sex	*n* (%)
Male	177 (50.28)
Female	175 (49.72)
Maternal education^a^	
Low	104 (29.55)
High	248 (70.45)
	Median [interquartile range]
PMA at scan, weeks	42.57 [41.29, 43.43]
GA at Birth, weeks	30.29 [28.14, 31.86]
Birth weight, grams	1300.00 [1010.00, 1622.00]
Age at assessment, months	20.04 [20.00, 20.16]
IMD rank	15422 [9310, 22772]
BSID-III cognitive development	95.00 [85.00, 105.00]
BSID-III language development	91.00 [79.00, 103.00]
BSID-III motor development	97.00 [91.00, 103.00]

^a^“Low” indicates exiting full-time education by 19 years old. “High” indicates exiting full-time education later than 19 years old or still in full-time education.

For TBM analysis, the 352 infants were subdivided into delayed and non-delayed groups on each subscale of the BSID-III (cognitive, language, and motor). For each subscale, we first compared voxel-wise whole-brain T2 images between the delayed and non-delayed groups with general linear modelling, including gestational age, PMA at scan, sex, and IMD as nuisance covariates (all scaled). In the second model, we included two additional binary nuisance variables (0: non-delay, 1: delay) representing delay in the other two BSID-III subscales to account for potential shared structural correlates across all three developmental domains. General linear modelling was performed using FSL Randomise (FMRIB Software Library, version 6.01) random permutation tests with 5000 permutations and threshold-free cluster enhancement [23–25]. Results were considered significant after family-wise error (FWE) correction.

## Results

### Comparisons of demographic information between groups with and without developmental delay

Table 1 shows the characteristics of the total sample, while Table 2 presents the characteristics of the various outcome groups. We also described the correlation among the three BSID-III subscales and the comparisons of the proportion of delayed cases among BSID-III subscales in Supplementary Appendix 3 (Supplementary Tables 1, 2). Participants with cognitive delay had a significantly lower gestational age and were more likely to live in more deprived neighbourhoods. Participants exhibiting language delays were predominantly male and were also more likely to live in more deprived neighbourhoods. Participants with motor delay had a significantly lower gestational age. Participants with developmental delays in one dimension were significantly more likely to have additional delays in the other two dimensions.

**Table 2:** Clinical, socio-demographic and behavioural characteristics of participants with delayed and non-delayed development (continued next page).

Variable	Cognitive development		
	Delay group (*n* = 68)	Typical group (*n* = 284)	Statistics
Sex			0.80 (0.05)
Male, *n* (%)	38 (55.88)	139 (48.94)	
Female, *n* (%)	30 (44.12)	145 (51.06)	
Maternal education			0.51 (0.05)
Low, *n* (%)	23 (33.82)	81 (28.52)	
High, *n* (%)	45 (66.18)	203 (71.48)	
PMA at scan, weeks, median [IQR]	42.64 [41.57, 43.86]	42.57 [41.14, 43.18]	10,777 (0.29)
GA at birth, weeks, median [IQR]	28.78 [27.25, 30.97]	30.57 [28.43, 32.00]	7072 (−1.14)***
Birth weight, gram, median [IQR]	1150.00 [912.50, 1402.75]	1325.00 [1055.00, 1663.75]	7110 (−190.00)***
Age at assessment, month, median [IQR]	20.03 [20.00, 20.14]	20.05 [20.00, 20.16]	8716 (−0.01)
IMD rank, median [IQR]	10,378.00 [6703.50, 18,240.00]	16,188.00 [9695.00, 23,084.50]	7086 (−4001.00)***
Cognitive development			
Delay, *n* (%)			
Typical, *n* (%)			
Language development			66.22 (0.43)***
Delay, *n* (%)	53 (77.94)	70 (24.65)	
Typical, *n* (%)	15 (22.06)	214 (75.35)	
Motor development			69.48 (0.46)***
Delay, *n* (%)	27 (39.71)	9 (3.17)	
Typical, *n* (%)	41 (60.29)	273 (96.13)	

Variable	Language development		
	Delay group (*n* = 123)	Typical group (*n* = 229)	Statistics
Sex			12.24 (0.19)***
Male, *n* (%)	78 (63.41)	99 (43.23)	
Female, *n* (%)	45 (36.59)	130 (56.77)	
Maternal education			0.60 (0.05)
Low, *n* (%)	40 (32.52)	64 (27.95)	
High, *n* (%)	83 (67.48)	165 (72.05)	
PMA at scan, weeks, median [IQR]	42.43 [41.22, 43.57]	42.57 [41.29, 43.29]	14,042 (0.00)
GA at birth, weeks, median [IQR]	30.57 [27.93, 32.00]	30.29 [28.29, 31.86]	14,163 (0.00)
Birth weight, gram, median [IQR]	1270.00 [1000.00, 1620.00]	1300.00 [1020.00, 1630.00]	13,590 (−25.00)
Age at assessment, month, median [IQR]	20.02 [20.00, 20.12]	20.06 [20.00, 20.16]	12,162 (−0.02)*
IMD rank, median [IQR]	12,758.00 [7241.00, 18,810.00]	16,898.00 [10,823.00, 24,590.00]	**10,522 (−3860.00)**
Cognitive development			66.22 (0.44)***
Delay, *n* (%)	53 (43.09)	15 (6.55)	
Typical, *n* (%)	70 (56.91)	214 (93.45)	
Language development			
Delay, *n* (%)			
Typical, *n* (%)			
Motor development			34.15 (0.32)***
Delay, *n* (%)	30 (24.39)	8 (3.49)	
Typical, *n* (%)	93 (75.61)	221 (96.51)	

Variable	Motor development		
	Delay group (*n* = 38)	Typical group (*n* = 314)	Statistics
Sex			1.36 (0.07)
Male, *n* (%)	23 (60.53)	154 (49.04)	
Female, *n* (%)	15 (39.47)	160 (50.96)	
Maternal education			0.73 (0.06)
Low, *n* (%)	14 (36.84)	90 (28.66)	
High, *n* (%)	24 (63.16)	224 (71.34)	
PMA at scan, weeks, median [IQR]	43.00 [41.89, 43.93]	42.57 [41.14, 43.29]	7532 (0.71)**
GA at birth, weeks, median [IQR]	28.57 [27.18, 29.93]	30.57 [28.29, 31.86]	4001 (−1.43)***
Birth weight, gram, median [IQR]	1110.00 [773.75, 1323.75]	1315.00 [1021.25, 1646.25]	4129 (−230.00)**
Age at assessment, month, median [IQR]	20.03 [20.00, 20.11]	20.04 [20.00, 20.17]	5446 (−0.01)
IMD rank, median [IQR]	14,684.50 [8476.00, 22,870.00]	15,688.00 [9425.75, 22,720.00]	5688 (−630.00)
Cognitive development			69.48 (0.46)***
Delay, *n* (%)	27 (71.05)	41 (13.06)	
Typical, *n* (%)	11 (28.95)	273 (86.94)	
Language development			34.15 (0.32)***
Delay, *n* (%)	8 (21.05)	93 (29.62)	
Typical, *n* (%)	30 (78.95)	221 (70.38)	
Motor development			
Delay, *n* (%)			
Typical, *n* (%)			

Mann–Whitney U test was used for continuous variables, the statistics are presented as U value (r value). Chi-squared test was used for categorical variables, the statistics are presented as Chi-square value (Cramer-V value), and the model had 1 degree of freedom. The values in bold represent statistically significant results (*P* < 0.05).

*IQR* Interquartile range.

^*^P < 0.05; **P < 0.01; ***P < 0.001.

### Comparisons of brain volumes between groups with and without developmental delay

**Fig. 1:**
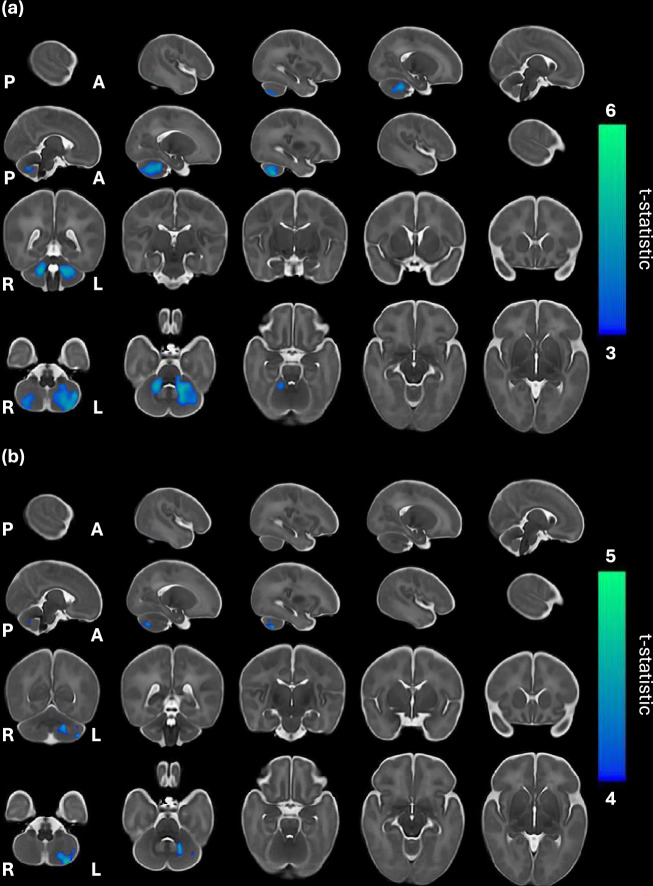
Volume difference at term between toddlers with and without delayed motor development. Map of T-statistic values of areas of significant reduction (blue-green) in volumes at term in toddlers with motor delayed development compared to non-delayed development (*pFWE* < 0.008) overlaid on study-specific brain template. Panel **a** adjusted for sex, PMA at scan, and IMD; Panel **b** adjusted for sex, PMA at scan, IMD, cognitive development, and language development. T-statistic range is shown on the colour bars. Anterior (A)-Posterior (P) and Left (L)-right (R) orientation follows radiological convention.

Firstly, we found no significant difference in brain volumes at term between toddlers with and without delayed cognitive (*t*_max_ = 4.044, *p*_FWE_ > 0.05) and language (*t*_max_ = 3.658, *p*_FWE_ > 0.05) development in whole-brain voxel-wise comparisons, after accounting for sex, PMA, and IMD. Toddlers with delayed motor development showed significantly reduced volumes in the cerebellum at term compared to those without delayed motor development (Fig. 1a).

Subsequently, given the high degree of overlap between participants with cognitive, language, and motor delays, we repeated the analyses accounting for selective BSID-III sub-scores. For instance, when investigating volumetric differences at term between toddlers with and without motor delay, we adjusted for sex, PMA at scan, IMD, and BSID-III cognitive and language sub-scores. Toddlers with delayed motor development displayed significantly reduced regional volumes in the left posterior lobe of cerebellum at term compared to those without delayed motor development (Fig. 1b). There was no significant difference in brain volumes at term between toddlers with and without delayed language development (*t*_max_ = 3.967, *p*_FWE_ > 0.05) as well as between those with and without delayed cognitive development (*t*_max_ = 3.800, *p*_FWE_ > 0.05).

## Discussion

This study examined structural brain alterations at term in very preterm toddlers with and without selective neurodevelopmental delays. Aligned with our first hypothesis, very preterm toddlers with motor delays displayed reduced bilateral cerebellar volumes at term. However, this effect was limited to the left posterior lobe of the cerebellum when accounting for cognitive and language developmental delays. These findings suggest that: (1) motor developmental delays in very preterm toddlers can be detected as early as the neonatal period using T2-weighted structural brain imaging, offering opportunities for earlier intervention; and (2) cognitive, language, and motor developmental delays may be associated with, albeit limited, overlapping structural brain alterations, hence the importance of treating outcomes as non-independent in statistical analyses.

Previous studies have extensively investigated the relationships between brain volumes at term and later developmental outcomes in very preterm children and have shown, for instance, that altered volumes in sensorimotor and premotor regions were associated with poorer cognitive and motor outcomes [8, 11]. Our previous research identified an association between brain lesions and motor impairments [26] and linked reduced neonatal volumes in salience network to poorer cognitive outcomes in childhood [15]. Expanding on these works, the current study explored how neonatal brain volume alterations related to neurodevelopmental outcomes in toddlerhood. Building upon prior studies focusing on individual developmental domains, we examined the unique association between each single domain and brain volumes, accounting for the potential neurostructural overlaps among the three domains.

Our findings of reduced left cerebellar volume at term in those toddlers with motor developmental delays can be interpreted in the context of previous literature showing altered cerebellar volume in preterm-born neonates compared to term-born controls [27, 28]. The third trimester of gestation, i.e., 24–40 weeks post-conception, is the most rapid and sensitive window for cerebellar development [29, 30]. The identification of reduced cerebellar volume associated with very preterm birth could be explained by impaired granule cell proliferation [31] or exposure to perinatal stressors [32]. Our findings further support the growing evidence that the cerebellum is susceptible to early extra-uterine influence and might be implicated in the aetiology of neurodevelopmental delay in toddlerhood [33].

Our findings of reduced left cerebellar volume at term in those toddlers with motor developmental delays are also consistent with previous literature showing that decreased cerebellar volume was associated with worse motor functioning in neonates and infants with cerebellar malformation and motor disorders [34, 35]. The potential mechanisms underlying the influence of altered cerebellar volume might include disrupted cerebro-cerebellar circuits essential for motor coordination, particularly the cortical-ponto-cerebellar loops modulated by cerebellar output [36, 37] and impaired olivocerebellar pathway development, potentially hindering the neural plasticity required for motor skill acquisition [38]. Given that infants with major brain lesions were excluded from the analysis, these volumetric differences are unlikely to reflect overt cerebellar injury and may be more plausibly associated with altered or delayed neurodevelopment. In our previous work, Vanes et al. also identified an association between reduced cerebellar volume at term and poorer psychomotor functioning in toddlerhood [39]. As such, altered cerebellar volume at term might be a potential biomarker for later motor delays and could be used to guide targeted, mechanism-based interventions. Nevertheless, it is important to note that not all VPT infants show adverse developmental outcomes or the same brain alterations despite shared prematurity. In our previous work, we identified subgroups of VPT children with distinct developmental outcomes. Notably, in some instances, no detectable neuroimaging differences were observed between children exhibiting typical development and those considered at higher risk. These findings suggest that factors extending beyond structural brain alterations may contribute to the observed heterogeneity in developmental outcomes [7]. Excluding infants with major brain lesions enables a more accurate characterisation of neurodevelopmental changes among low-risk very preterm children whose developmental profiles are less likely to be confounded by severe neurological injury. Additionally, socioeconomic advantages, enriched home environments, and early access to supportive interventions have been shown to promote resilience by modulating neural activity and supporting compensatory pathways in development [15, 39]. Finally, the high plasticity and network-level adaptability of the developing brain mean that subtle structural variations may not necessarily translate into functional difficulties [10, 40]. Together, these considerations highlight that developmental outcomes in VPT infants arise from a complex interaction of biological vulnerability, environmental context, and individual differences in neuroplasticity.

In contrast, we found no volumetric differences at term between toddlers with and without cognitive or language delays. These findings  are consistent with prior evidence suggesting heterochronicity in brain–behaviour associations, whereby cognitive and language functions rely on later maturing cortices, predominantly prefrontal and temporal regions, where alterations may not be sufficiently evident at term to correlate with subsequent outcomes [41, 42]. The absence of domain-specific associations in these areas may reflect the limited sensitivity of early brain structural markers for functions that emerge more fully over time (i.e., cognitive and language). By comparison, motor development in early life is more directly linked to cerebellar maturation, which is already underway at term and thus more readily captured by volumetric measures at this stage. However, after accounting for language and cognitive delays, the differences between toddlers with and without motor delays in cerebellar volume at term became less extensive. These findings suggest that cerebellar spatial compartments may be engaged across multiple behavioural domains, reflecting its function as a central integrative structure [43]. They also highlight the role of the posterior cerebellar lobe in supporting motor function during early human development [44].

Despite the strengths of this study, several limitations must be acknowledged. Firstly, we employed a categorical approach to define developmental delay based on established screening thresholds. Whilst this strategy enhances utility for identifying atypicality and facilitates risk stratification, binarising neurodevelopmental scores may reduce statistical power relative to continuous correlational analyses. Evidence also suggests that many infants move between ‘delayed’ and ‘non-delay’ categories during the first two years of life [45]. This indicates that the instability of developmental status may skew categorical group results. Consequently, the neurobiological markers we discovered linked to developmental delays should be interpreted with caution. Secondly, the accuracy of TBM is fundamentally dependent on the quality of the registration. While we implemented a validated pipeline and rigorous manual quality checks, we cannot entirely exclude the possibility that suboptimal registration, rather than biological variability, may influence registration performance and potentially bias results. Thirdly, while the BSID-III is a widely recognised assessment tool for early development, evidence supporting its ability to accurately identify developmental delays in high-risk populations more specifically is still scarce. Recent studies indicate that this tool may underestimate developmental delays in very preterm infants [46, 47].

By leveraging various dimensions of neurodevelopment in toddlerhood, we were able to further explore the early structural underpinnings of delayed motor, cognitive, and language development in very preterm infants. Future research can further explore other factors (including parenting, environmental, and genetic information) that might influence the relation between brain structure at term and developmental outcomes in toddlerhood. Future studies should incorporate a broader range of biological, genetic, and perinatal risk factors to more thoroughly elucidate the potential mechanisms underlying brain structure and neurodevelopmental trajectories. Additional BSID-III subscales, including fine and gross motor domains, could be employed to further elucidate the relationship between brain structure and specific facets of neurodevelopment. Moreover, research has highlighted the significant influence of sex on neurodevelopment [48] and future studies may investigate the sex-specific brain structural underpinnings of neurodevelopment in males and females, rather than treating sex as a confounding variable.

To summarise, this study identified volumetric alterations in the cerebellum, a critical region for motor processing, in very preterm infants with delayed motor development in toddlerhood compared to those without. By advancing our understanding of neonatal structural alterations and their association with different aspects of early development, these findings may inform early screening for developmental delays and support  the development of targeted early interventions to improve infants’ outcomes.

## Supplementary information

Supplementary materials

## Data Availability

Available to referees at submission and to readers promptly upon request.
