# Supplementary material for: Associations between neonatal brain structure and neurodevelopmental outcomes following very preterm birth
Source: J Perinatol. 2026 Apr 15;46(7):1259–66. doi: 10.1038/s41372-026-02672-3 (PMC13423810; doi:10.1038/s41372-026-02672-3)
Supplement: Supplementary file 1 — Supplementary materials [file 41372_2026_2672_MOESM1_ESM.docx]

**Associations Between Neonatal Brain Structure and Neurodevelopmental Outcomes Following Very Preterm Birth**

**Appendix 1: Methods supplementary information**

**MRI acquisition**

Initial quality control for the imaging analysis began with 485 successful T2-weighted scans. Participants with visually detectable artefacts (N=10) and major brain lesions (N=40) were excluded. Those scanned at a PMA of 45 weeks or greater (N=50) were also excluded to minimise the risk of registration failures. Following these clinical and technical exclusions, two additional subjects were removed due to processing failures. Finally, 31 participants were excluded from the imaging analysis due to incomplete neurodevelopmental assessments.

Qualitative MRI classification was performed by a senior radiographer, resulting in three categories: major lesions, defined as cystic periventricular leukomalacia, more than ten punctate white matter lesions, and/or grade 3 or 4 germinal matrix haemorrhage; minor lesions, defined as any other lesions; and no lesions (Barnett et al., 2018).

**MRI processing**

Image processing was performed using a previously validated neonatal pipeline (Makropoulos et al., 2014), which included initial field bias correction and subsequent tissue type segmentation. A study-specific template was generated from a subset of 161 participants using Advanced Normalization Tools (ANTS) software (Avants et al., 2008). This customised template was successfully used in our previous work (Ball et al., 2017; Vanes et al., 2021).

Individual images were subsequently registered to this study-specific template using the multimodal Symmetric Normalisation (SyN) algorithm, also within the ANTS framework (Avants et al., 2008). To maximise the accuracy of the spatial registration, both the T2-weighted images and the corresponding T2-based tissue type segmentation masks were used as complementary input modalities (Makropoulos et al., 2018).

Deformation tensor fields (i.e., warps) resulting from the non-linear registration process were used to quantify local changes in brain volume. Specifically, we calculated the logarithm of the Jacobian determinant (log Jacobian) maps (Avants & Gee, 2004). This measure reflects the local expansion (positive values) or shrinkage (negative values) of each voxel's volume relative to the common template space. Log Jacobian maps were spatially smoothed with a Gaussian kernel of 4mm full width at half maximum. In order to reduce computational load, maps were down-sampled to 2mm isotropic resolution. Finally, we included only voxels falling within the boundaries of the brain tissue. This mask was defined using the neonatal adaptation of the Automated Anatomical Labelling (AAL) atlas (Shi et al., 2011; Tzourio-Mazoyer et al., 2002), resulting in a final analysis space of 37,947 voxels.

**Appendix 2: Supplementary Figures**


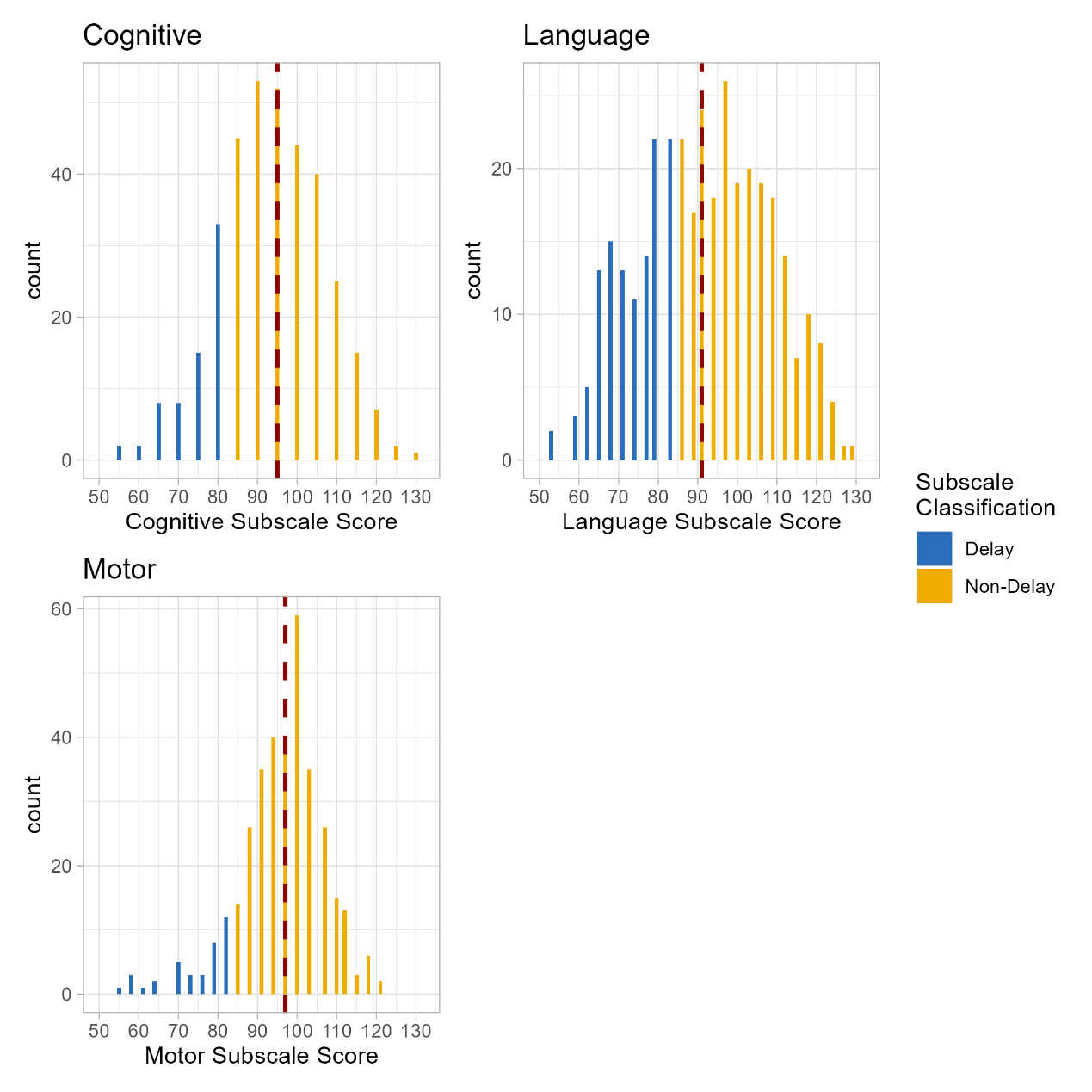


**Figure S1: Distribution of BSID-III Cognitive, Language, and Motor Subscale Scores.** *Different developmental status was classified by colour: Delay (blue) vs. No Delay (yellow). The dashed red vertical line indicates the median score for each subscale.*

**
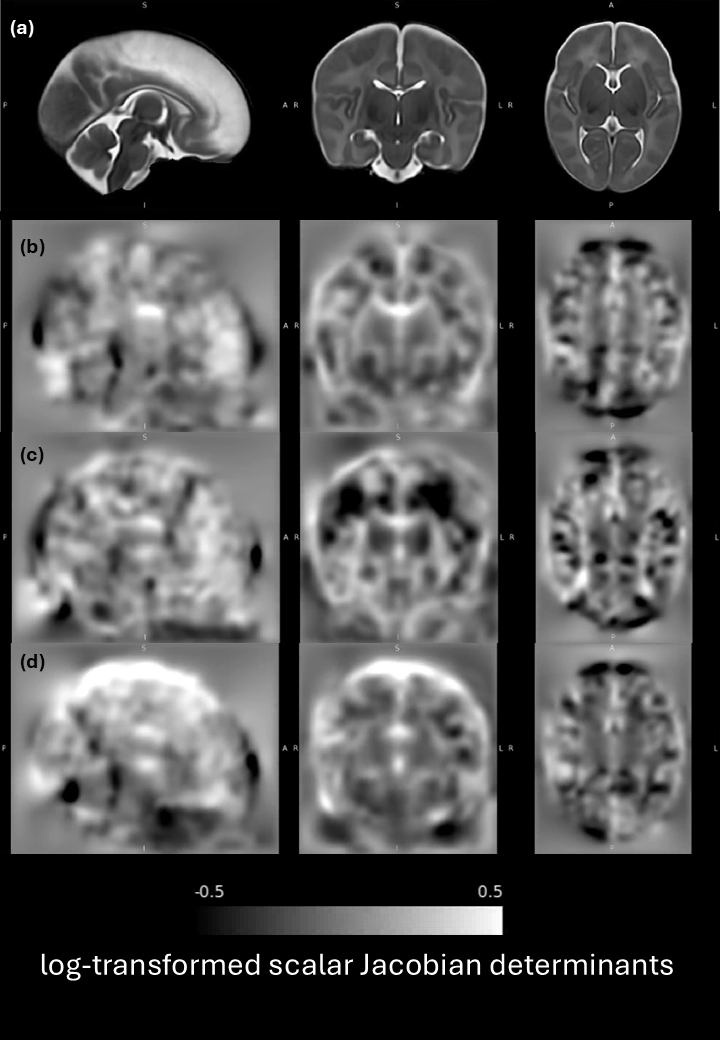
Figure S2: Whole-brain study template (a) and participants’ T2-weighted image registered to the template (b-d).**


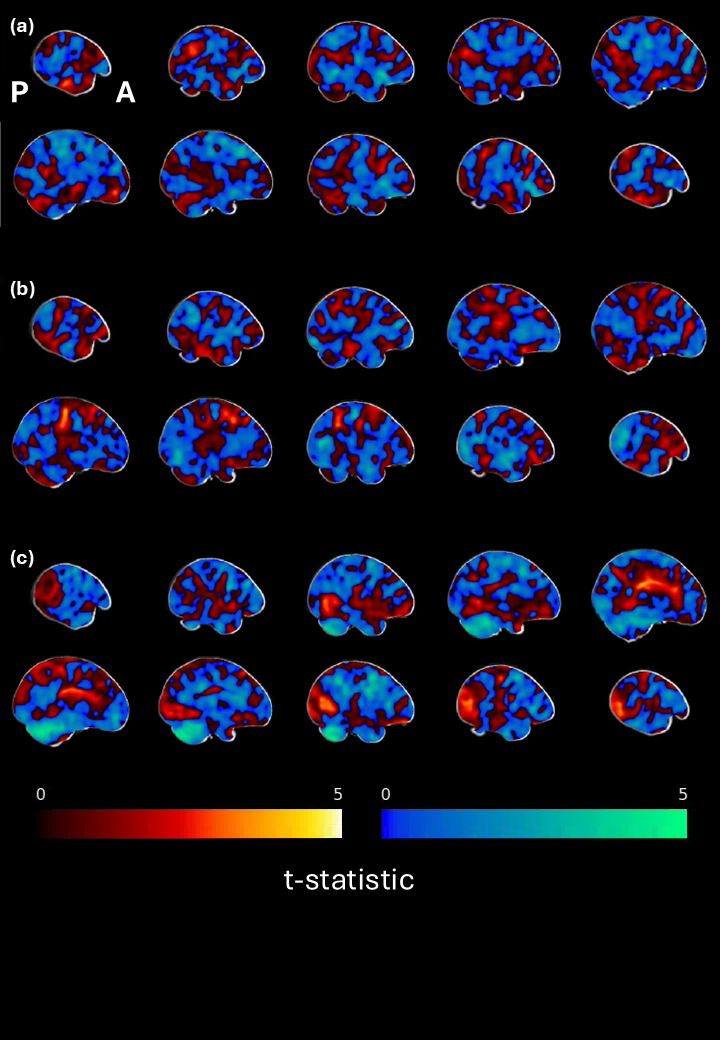
**Figure S3: Unthresholded volume difference at term between toddlers with and without delayed cognitive (a), language (b) and motor (c) development.**

*Map of T-statistic values of areas of reductions (blue-green) and increases (red-yellow) in volumes at term in toddlers with delayed development compared to non-delayed development, overlaid on the study-specific brain template. Panel adjusted for sex, PMA at scan, IMD and delays in the other two developmental domains (i.e., language and motor for Panel a). The non-threshold T-statistic range is shown on the colour bars.*


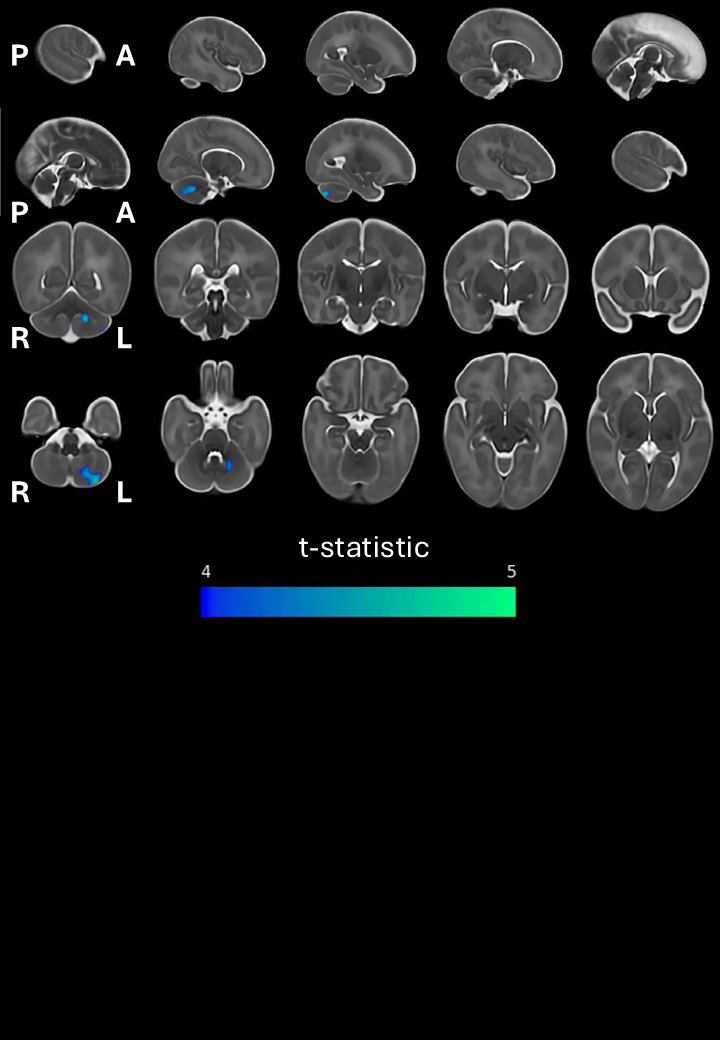


**Figure S4: Significant volume difference at term between toddlers with and without delayed motor development, additionally adjusting for GA.**

*Map of T-statistic values of areas of significant reductions (blue-green) in volumes at term in toddlers with motor delayed development compared to non-delayed development (family-wise-error-corrected p < 0.008) overlaid on study-specific brain template, adjusted for sex, PMA at scan, GA, IMD, cognitive and language development. T-statistic range is shown on the colour bars.*

**Appendix 3: Supplementary Tables**

**Table 1: Correlation analysis among BSID-III Cognitive, Language, and Motor Subscale Scores.**

| Correlation | R value [95% CI] |
| --- | --- |
| Cognitive-Language | 0.67 [0.61, 0.73]*** |
| Cognitive-Motor | 0.63 [0.56, 0.69]*** |
| Language-Motor | 0.61 [0.54, 0.67]*** |

*:P<0.05; **:P<0.01;***:P<0.001

**Table 2: Comparisons of Proportions of Delayed Cases among BSID-III Cognitive, Language, and Motor Subscales.**

| Comparisons | Statistics | |
| --- | --- | --- |
|  | Q value (degree of freedom)^a^ | χ^2^ (degree of freedom)^b^ |
| Cognitive-Language | 93.70 (2)*** | 34.31 (1)*** |
| Cognitive-Motor |  | 16.17 (1)*** |
| Language-Motor |  | 69.86 (1)*** |

*:P<0.05; **:P<0.01;***:P<0.001. ^a^Cochran's Q test; ^b^McNemar's Chi-Square test, all p values were Bonferroni-adjusted for multiple comparisons.

**Reference**

Avants, B., & Gee, J. C. (2004). Geodesic estimation for large deformation anatomical shape averaging and interpolation. *Neuroimage*, *23 Suppl 1*, S139-150. <https://doi.org/10.1016/j.neuroimage.2004.07.010>

Avants, B. B., Epstein, C. L., Grossman, M., & Gee, J. C. (2008). Symmetric diffeomorphic image registration with cross-correlation: evaluating automated labeling of elderly and neurodegenerative brain. *Med Image Anal*, *12*(1), 26-41. <https://doi.org/10.1016/j.media.2007.06.004>

Ball, G., Aljabar, P., Nongena, P., Kennea, N., Gonzalez-Cinca, N., Falconer, S., Chew, A. T. M., Harper, N., Wurie, J., Rutherford, M. A., Counsell, S. J., & Edwards, A. D. (2017). Multimodal image analysis of clinical influences on preterm brain development. *Ann Neurol*, *82*(2), 233-246. <https://doi.org/10.1002/ana.24995>

Barnett, M. L., Tusor, N., Ball, G., Chew, A., Falconer, S., Aljabar, P., Kimpton, J. A., Kennea, N., Rutherford, M., David Edwards, A., & Counsell, S. J. (2018). Exploring the multiple-hit hypothesis of preterm white matter damage using diffusion MRI. *Neuroimage Clin*, *17*, 596-606. <https://doi.org/10.1016/j.nicl.2017.11.017>

Makropoulos, A., Gousias, I. S., Ledig, C., Aljabar, P., Serag, A., Hajnal, J. V., Edwards, A. D., Counsell, S. J., & Rueckert, D. (2014). Automatic whole brain MRI segmentation of the developing neonatal brain. *IEEE Trans Med Imaging*, *33*(9), 1818-1831. <https://doi.org/10.1109/TMI.2014.2322280>

Makropoulos, A., Robinson, E. C., Schuh, A., Wright, R., Fitzgibbon, S., Bozek, J., Counsell, S. J., Steinweg, J., Vecchiato, K., Passerat-Palmbach, J., Lenz, G., Mortari, F., Tenev, T., Duff, E. P., Bastiani, M., Cordero-Grande, L., Hughes, E., Tusor, N., Tournier, J. D.,…Rueckert, D. (2018). The developing human connectome project: A minimal processing pipeline for neonatal cortical surface reconstruction. *Neuroimage*, *173*, 88-112. <https://doi.org/10.1016/j.neuroimage.2018.01.054>

Shi, F., Yap, P. T., Wu, G., Jia, H., Gilmore, J. H., Lin, W., & Shen, D. (2011). Infant brain atlases from neonates to 1- and 2-year-olds. *PLoS One*, *6*(4), e18746. <https://doi.org/10.1371/journal.pone.0018746>

Tzourio-Mazoyer, N., Landeau, B., Papathanassiou, D., Crivello, F., Etard, O., Delcroix, N., Mazoyer, B., & Joliot, M. (2002). Automated anatomical labeling of activations in SPM using a macroscopic anatomical parcellation of the MNI MRI single-subject brain. *Neuroimage*, *15*(1), 273-289. <https://doi.org/10.1006/nimg.2001.0978>

Vanes, L. D., Hadaya, L., Kanel, D., Falconer, S., Ball, G., Batalle, D., Counsell, S. J., Edwards, A. D., & Nosarti, C. (2021). Associations Between Neonatal Brain Structure, the Home Environment, and Childhood Outcomes Following Very Preterm Birth. *Biol Psychiatry Glob Open Sci*, *1*(2), 146-155. <https://doi.org/10.1016/j.bpsgos.2021.05.002>
